# Supplementary material for: Unconventional Treatments for Pancreatic Cancer: A Systematic Review
Source: Cancers (Basel). 2025 Apr 25;17(9):1437. doi: 10.3390/cancers17091437 (PMC12071172; doi:10.3390/cancers17091437)
Supplement: Supplementary file 1 [file cancers-17-01437-s001.zip › File S1.pdf]

File S1

A table of excluded studies for full-text review is presented.

Author	Year	Study Design	Reason for Exclusion	Comments
Parsons et al.	2016	Retrospective case–control study	Insufficient data reported and study design	
Morag et al.	2021	Case report	Study design	Case report of a patient with metastatic pancreatic cancer and testicular/groin pain treated with acupuncture in abdominal wall muscle trigger point.
Meng et al.	2009	Prospective single cohort study	Study design, outcome assessed and insufficient data reported	Prospective phase I trial aimed at evaluating the tolerability of huachansu in patients with stage III or IV hepatocellular carcinoma, non-small-cell lung cancer, or pancreatic cancer. Only two patients with pancreatic cancer were enrolled; data regarding overall survival were not reported.
Ikemoto et al.	2014	Prospective study	Insufficient data and outcomes assessed	
Bonucci et al.	2018	Case report	Study design	Case report of two patients with metastatic pancreatic cancer who were treated using an integrated cancer treatment approach combining chemotherapy with natural remedies, extracts, and hyperthermia. Both patients had a notable remission of primary and metastatic lesions.
Gonzalez et al.	1999	Case series	Study design	
Liu et al.	2023	Case report	Study design	
Ramasamy et al.	2018	Case report	Study design	
Wode et al.	2020	Protocol	Study design	Protocol of the study conducted by Wode et al. (2024).
Werthmann et al.	2018	Case report	Study design	
Stern et al.	2019	Case report	Study design	
Ritter et al.	2010	Case report	Study design	
Jiang et al.	2019	Protocol	Study design	
Hua Qiang et al.	2015	Retrospective	Non-English article	
Shen et al.	2012	Not reported	Non-English article	

Zhang et al.	2017	Not reported	Non-English article	
Shi et al.	2005	Not reported	Non-English article	
Wang et al.	2020	Not reported	Non-English article	
Sakata et al.	1994	Not reported	Full text not available	
Eggers et al.	2023	Not reported	Full text not available	
Troger et al.	2013	Randomized controlled trial	Study design	Interim analysis of the study conducted by Troger et al. (2014).
Werthmann et al.	2018	Case report	Study design	
Stie et al.	2021	Protocol	Study design	
Kranz et al.	2008	Case report	Study design	
Saif et al.	2010	Prospective cohort study	Insufficient data and outcomes assessed	
Hashimoto et al.	2021	Retrospective	Insufficient data and outcomes assessed	
Hashimoto et al.	2022	Protocol	Study design	
Matthes et al.	2008	Case series	Old study and non-English article	
Friess et al.	1996	Case series	Full text not available	
Schaefermeyer	1998	Retrospective	Old study	
Hecht JR et al.	2003	Prospective cohort study	Old study	Prospective cohort study on intratumor EUS-guided injections of ONYX-015 (adenovirus) in patients with unresectable pancreatic cancer.
Mulvihill S et al.	2001	Prospective cohort study	Old study	Prospective cohort study on intratumoral injection of an E1B-55 kDa region-deleted adenovirus into primary pancreatic tumors in patients with locally advanced pancreatic cancer.
Hirooka Y et al.	2018	Prospective cohort study	Type of treatment	Phase I trial aimed to evaluate the feasibility and efficacy of direct injection of HF10 into unresectable locally advanced pancreatic cancer under endoscopic ultrasound (EUS) guidance in combination with erlotinib and gemcitabine administration.

Navneet Dhillon et al.	2008	Prospective cohort study	Old study
