# Supplementary material for: Unconventional Treatments for Pancreatic Cancer: A Systematic Review
Source: Cancers (Basel). 2025 Apr 25;17(9):1437. doi: 10.3390/cancers17091437 (PMC12071172; doi:10.3390/cancers17091437)
Supplement: Supplementary file 1 [file cancers-17-01437-s001.zip › supplementary table.pdf]

Unconventional treatments for pancreatic cancer: a systematic review

Author	Trial registration number	Sponsorship or funding	Blinding
**Studies evaluating the effect of Chinese herbal medicine**			
Wong et al [9]	NA	No	not applicable
Saif et al [20]	NA	Yes: National Comprehensive Cancer Network (NCCN) from general research support provided by Roche Laboratories;	not applicable
Lee et al [11]	NA	not reported	not applicable
Meng et al [10]	Yes [NCT00837239 clinicaltrials.gov]	Yes: National Institute of Health grant U19CA121503-01.	Yes
Kuo et al [21]	NA	Yes: China Medical University and Taiwan Ministry of Health and Welfare Clinical Trial and Research Center of Excellence (MOHW106-TDU-B-212-113004).	
Li et al [22]	NA	No	not applicable
Xue Yang et al [12]	NA	Yes: National Science Foundation of China (nos. 81173376 and 81473441)	not applicable
Cao et al [13]	NA	Yes: Shanghai Municipal Hospital New Developing and Leading Edge Technology Project (SHDC12010120) and Shanghai Health System Excellent Subject Leader Developing Plan	
Ouyang et al [14]	NA	Yes: National Cancer Institute, Ministry of Science and Technology, BClimbing Up (Project of Shanghai Municipal Commission for Science and Technology, no. 064307053; by Shanghai Nature Science Fund no. 09ZR1406800) and by Shanghai Municipal Health Bureau	NA
Song et al [15]	NA	no	NA
**Studies evaluating the effect of mistletoe extract**			
Schad et al [23]	NA	Yes: The NO database project was funded by the Software AG Stiftung Darmstadt, Weleda AG, Arlesheim, Abnoba GmbH, Pforzheim, and Helixor GmbH, Rosenfels, Germany.	not applicable
Thronicke et al [24]	NA	No	not applicable
Troger et al [25]	Yes (ISRCTN70760582 Current Controlled Trials database)	Yes: Swiss Cancer Research Association	No
Wode et al [27]	Yes (EudraCT 2014–004552–64, NCT02948309)	Yes: Department of Oncology Endowment Fund at Karolinska University Hospital, Cancer Research Funds of Radiumhemmet, Gyllenberg Foundation, Ekhaga Foundation, Dagmar Ferbs Memorial Fund, Cancer Research Foundation in Northern Sweden, The Sjöberg Foundation. Iscador AG, Switzerland, manufactured and supplied both ME and placebo and post-trial ME free of charge.	Yes
Axtner et al [26]	NA	Yes: Software AG Stiftung Darmstadt -Germany, Weleda AG Arlesheim -Switzerland, Abnoba GmbH Pforzheim -Germany, and Helixor GmbH Rosenfels -Germany	not applicable
**Studies evaluating the effect of curcumin**			
Pastorelli et al [28]	NA	No	not applicable

Kanai et al [16]	Yes (University Hospital Medical Information Network (UMIN) Clinical Trial Registry (ID 000001386)).	Yes: the work was supported by the Grant-in-Aid for Young Scientists (21790661) from the Japan Society for the Promotion of Science, Japanese Research Foundation for Clinical Pharmacology and Smoking Research Foundation.	not applicable
Kanai et al [17]	Yes (University Hospital Med- ical Information Network Clinical Trials Registry 000002950)	Yes: Grant-in-Aid from the Japan Society for the Promotion of Science (24590655) and the Japanese Research Foun-dation for Clinical Pharmacology.	not applicable
Epelbaum et al [29]	NA	No	not applicable
**Studies evaluating unconventional therapies for pancreatic cancer pain**			
Chen et al [18]	NA	Yes: national Natural Science Foundation of China (NSFC) (81202751 to Le Kuai), and Shanghai Science and Technology Committee	Yes
Tian et al [19]	NA	Yes: This study was supported by the College Foundation of Fudan University Shanghai Cancer Center (HL201304).	Not reported

Supplementary table S1: quality of included studies.
